# Increased plasma levels of lncRNA H19 and LIPCAR are associated with increased risk of coronary artery disease in a Chinese population

**DOI:** 10.1038/s41598-017-07611-z

**Published:** 2017-08-08

**Authors:** Zhen Zhang, Wei Gao, Qing-Qing Long, Jian Zhang, Ya-Fei Li, Dong-Chen liu, Jian-Jun Yan, Zhi-Jian Yang, Lian-Sheng Wang

**Affiliations:** 1 Department of Cardiology, The First Affiliated Hospital of Nanjing Medical University, Nanjing, 210029 China; 2 Department of Geriatrics, Sir Run Run Hospital, Nanjing Medical University, Nanjing, 211166 China; 3 Key Laboratory for Aging & Disease, Nanjing Medical University, Nanjing, 211166 China

## Abstract

Recent studies in animal models and humans show that long non-coding RNAs (lncRNAs) are involved in the development of atherosclerosis, which contributes to the pathological foundation of coronary artery disease (CAD). LncRNAs in plasma and serum have been considered as promising novel biomarkers for diagnosis and prognosis of cardiovascular diseases, especially CAD. We here measured the circulating levels of 8 individual lncRNAs which are known to be relevant to atherosclerosis in the plasma samples from 300 patients with CAD and 180 control subjects by using quantitative real-time reverse transcription-polymerase chain reaction (qRT-PCR) methods. We found that the plasma level of H19 and long intergenic non-coding RNA predicting cardiac remodeling (LIPCAR) were significantly increased in patients with CAD. The area under the receiver operating characteristic curve was 0.631 for H19 and 0.722 for LIPCAR. Multivariate logistic regression analyses indicated that plasma H19 and LIPCAR were independent predictors for CAD, even after adjustment for traditional cardiovascular risk factors. Further studies identified that plasma levels of H19 and LIPCAR were also increased in CAD patients with heart failure compared to those with normal cardiac function. Taken together, our results suggest that increased plasma levels of H19 and LIPCAR are associated with increased risk of CAD and may be considered as novel biomarkers for CAD.

## Introduction

Coronary artery disease (CAD) remains one of the major causes of mortality and morbidity in many countries, including China^1^. Numerous studies have identified several risk factors for CAD, including hypertension, dyslipidaemia, diabetes, obesity, smoking, dietary, gender, *etc*.^2^. Recently, genomics researches have revealed a series of new candidate biomarkers that may contribute to the pathogenesis of CAD^3^.

Long non-coding RNAs (lncRNAs), a novel class of non-coding RNAs, are defined as transcripts that are longer than 200 nt and lacking protein-encoding capacity^4^. LncRNAs play crucial roles in chromatin modification, imprinting, cell differentiation and proliferation, transcription, translation and other important biological processes^5^. Atherosclerosis, the main pathophysiological cause of CAD, is initiated by endothelial injury and activation, which leads to infiltration and proliferation of vascular smooth muscle cells (VSMC), leukocytes and other inflammatory cells in the arterial wall^6^. Recently, lncRNAs have emerged as important regulators in various pathological processes that contribute to the development of atherosclerosis^7–9^. For instance, the lincRNA metastasis associated lung adenocarcinoma transcript 1 (MALAT1) regulates blood vessel growth and MALAT1 inhibition prevents human endothelial cell proliferation and reduces vascular growth^10^. LncRNA H19 is highly expressed in the neo-intima after injuries and in human atherosclerotic lesions, but barely expressed in normal coronary arteries^11, 12^. LncRNA highly upregulated in liver cancer (HULC) and Apolipoprotein A1 antisense (APOA1-AS) can modulate multiple key lipometabolism-related genes and play important roles in lipid homeostasis^13, 14^. TNFα and hnRNPL related immunoregulatory LincRNA (THRIL) can promote the transcriptional process of TNF-α and induce inflammation^15^. By using microarray analysis in a rat model of ischemic heart disease, we also identified 331 pairs of differentially expressed lncRNAs and nearby coding genes, indicating that lncRNA might be also involved in the pathogenesis of CAD^16^.

To date, non-coding RNAs, including microRNAs and lncRNAs, in plasma and serum have been considered as promising novel biomarkers for diagnosis and prognosis of cardiovascular diseases^17^. We have previously demonstrated that lipometabolism-related microRNA-122 and microRNA-370 were associated with the risk and severity of CAD^18^. However, studies on circulating lncRNAs for the risk of CAD remain sparse. In the present study, we examined plasma levels of eight cardiac-related or atherosclerosis-related lncRNAs, including H19, long intergenic non-coding RNA predicting cardiac remodeling (LIPCAR), APOA1-AS, THRIL, HULC, SLC26A4-AS1, LincRNA-Cox2, LincRNA-p21^11–15, 19–22^, in patients with angiographically demonstrated CAD to investigate the possibility of these circulating lncRNAs as novel biomarkers for CAD.

## Materials and Methods

### Study subjects

From 2014 to 2016, 480 consecutive subjects (296 males and 184 females), aged 42–78 years, who underwent coronary angiography for suspected or known coronary atherosclerosis at the First Affiliated Hospital of Nanjing Medical University in China were enrolled in this study. Coronary artery disease (CAD) was defined as angiographic evidence of at least one segment of a major coronary artery, including the left anterior descending, left circumflex, or right coronary artery, with >50% organic stenosis. The severity of CAD was assessed by the Gensini score system based on the degree of luminal narrowing and its geographic importance^23^. Subjects with normal coronary arteries were considered as controls. Two cardiologists who were unaware of the patients included in this study assessed the angiograms. All subjects included in this study had no family history of CAD and no history of significant concomitant diseases, including hepatic failure, renal failure, hepatitis, cardiomyopathy, congenital heart disease, bleeding disorders, previous thoracic irradiation therapy, and malignant diseases. CAD patients were divided into three subgroups: stable angina pectoris (SAP), unstable angina pectoris (UAP), and acute myocardial infarction (AMI), which were defined as previously described^24^. The diagnosis of chronic heart failure (CHF) was made on the basis of typical symptoms and signs, and evidence of left ventricular enlargement and systolic functional impairment by echocardiography, according to the American College of Cardiology/American Heart Association guidelines^25^. Hypertension was defined as resting systolic blood pressure (SBP) above 140 mmHg and/or diastolic blood pressure (DBP) above 90 mmHg or in the presence of active treatment with antihypertensive agents. Diabetes mellitus was defined as fasting blood glucose (FBG) >7.0 mmol/L or a diagnosis with diet adjustment or anti-diabetic drug therapy. Smoking was defined as >10 cigarettes per day. Written informed consent was obtained from each participant and this study was approved by the Ethics Committee of the First Affiliated Hospital of Nanjing Medical University. All of the experiments in the present study were performed in accordance with the relevant approved guidelines and regulations.

### Laboratory measurements

Fasting blood sample was collected from each subject and anticoagulated with ethylenediamine tetraacetic acid (EDTA) dipotassium salt in the early morning. The sample was separated immediately by centrifugation at 3000 g for 15 min at 4 °C to retrieve plasma. The plasma was then stored at −80 °C until assayed. Total cholesterol (TC), triglyceride (TG), high-density lipoprotein cholesterol (HDL-C), low-density lipoprotein cholesterol (LDL-C) levels were measured enzymatically on a chemistry analyzer (Olympus Au2700, First Chemical Ltd., Tokyo, Japan). Glucose levels were measured by a glucose oxidase method (Reagent kit; Diagnostic Chemicals Ltd., Oxford, CT, UK).

### RNA extraction and reverse transcription (RT)

Total RNA was isolated from 400 μL of plasma using the mirVanaTM PARISTM Kit (Ambion, Austin, TX) according to the manufacturer’s instructions with modification. For normalization of sample-to-sample variation, 25 fmol of synthetic C.elegans miRNA cel-miR-39 (Qiagen, Germany) was added to each sample after addition of 2 × Denaturing Solution (Ambion, Austin, TX) [24]. RNA was dissolved in 100 μL of RNase-free water, and then stored at −80 °C until analysis. Total RNA was reverse transcribed using the DRR037A PrimeScript® RT Master mix (Takara, Dalian, P.R. China). The RT reaction was incubated at 37 °C for 15 min, at 85 °C for 5 s, and then held at 4 °C. The cDNA product was stored at −20 °C until analysis.

### Quantitative real-time PCR (qRT-PCR)

To detect plasma levels of lncRNA, 2 μL of the cDNA product was used as template in 10 μL reaction containing 5 μL of TaqMan® Universal PCR Master Mix (Applied Biosystems, Foster, CA), 1 μL of specific primer (Supplementary Table 1), 2 μL of RNase-free water. qRT-PCR was performed with 7900HT real-time PCR system (Applied Biosystems, Foster, CA) at 95 °C for 10 min, followed by 40 cycles of 95 °C for 15 s and 60 °C for 1 min. Triplicate measurements were obtained for each sample on a 384-well plate. Data were analyzed with SDS Relative Quantification Software version 2.2.2 (Applied Biosystems, Foster, CA), with the automatic Ct setting for assigning baseline and threshold for Ct determination. The relative expression level of each individual lncRNA after normalization to cel-miR-39 was calculated using the 2^−ΔΔCt^ method.

### Statistical analysis

Normality of distribution was assessed using the Kolmogorov-Smirnov test. Comparison between two groups was performed with Student’s t tests or Mann–Whitney U tests. For comparison among more than 2 groups, one-way ANOVA or the Kruskal–Wallis test was used as appropriate. Pearson χ2 test was used to compare qualitative variables represented as frequencies. The correlations between plasma levels of lncRNAs and other variables were calculated using Spearman correlation coefficient. Univariate analysis and multi-variate logistic regression analysis were undertaken to determine the variables that independently contributed to the presence of CAD. Odds ratio (OR) and 95% confidence interval (CI) were also calculated. All tests were two-sided and *P* < 0.05 was considered statistically significant. Receiver operating characteristic (ROC) curve and the area under ROC curve (AUC) were used to assess the sensitivity and specificity of lncRNA as a novel diagnostic tool for the detection of CAD.

## Results

### Characteristics of study subjects

Table 1 presents the characteristics of the study population. A total of 300 subjects with coronary artery disease (CAD) and 180 controls were enrolled in the study. Compared with the controls, patients with CAD had higher levels body mass index (BMI), TC, LDL-C, prevalence of hypertension, but lower HDL-C. No significant difference was found in age, gender, smoking, diabetes, creatinine, TG, and fasting blood glucose (FBG). The CAD group consists of 81 patients with stable angina pectoris (SAP), 189 patients with unstable angina pectoris (UAP), and 30 patients with acute myocardial infarction (AMI).

**Table 1:** Characteristics of study subjects.

Characteristics	CAD (n = 300)	Control (n = 180)	*P* value
Age (years)	64.21 ± 10.77	63.22 ± 10.07	0.303
Gender (F/M)	112/188	72/108	0.561
BMI	25.08 ± 2.83	24.28 ± 3.24	0.007
Smoking, n (%)	137 (45.7%)	87 (48.3%)	0.571
TC (mmol/L)	4.72 ± 1.09	4.26 ± 1.08	<0.001
HDL-C (mmol/L)	1.07 ± 0.27	1.21 ± 0.31	<0.001
LDL-C (mmol/L)	3.13 ± 0.77	2.75 ± 0.81	<0.001
TG (mmol/L)	1.51 ± 0.98	1.35 ± 0.75	0.077
FBG (mmol/L)	5.58 ± 2.04	5.32 ± 0.77	0.067
Creatinine (μmol/L)	72.88 ± 18.31	72.33 ± 21.15	0.783
Hypertension, n (%)	193 (64.3%)	52 (28.9%)	<0.001
Diabetes, n (%)	56 (18.7%)	32 (17.9%)	0.807
SAP, n (%)	81 (27%)	—	—
UAP, n (%)	189 (63%)	—	—
AMI, n (%)	30 (10%)	—	—

CAD, coronary artery disease; BMI, body mass index; TC, total cholesterol; HDL-C, high-density lipoprotein cholesterol; LDL-C, low-density lipoprotein cholesterol; TG, triglyceride; FBG, fasting blood glucose; SAP, stable angina pectoris; UAP, unstable angina pectoris; AMI, acute myocardial infarction.

### Plasma levels of H19 and LIPCAR are increased in CAD patients

**Figure 1:**
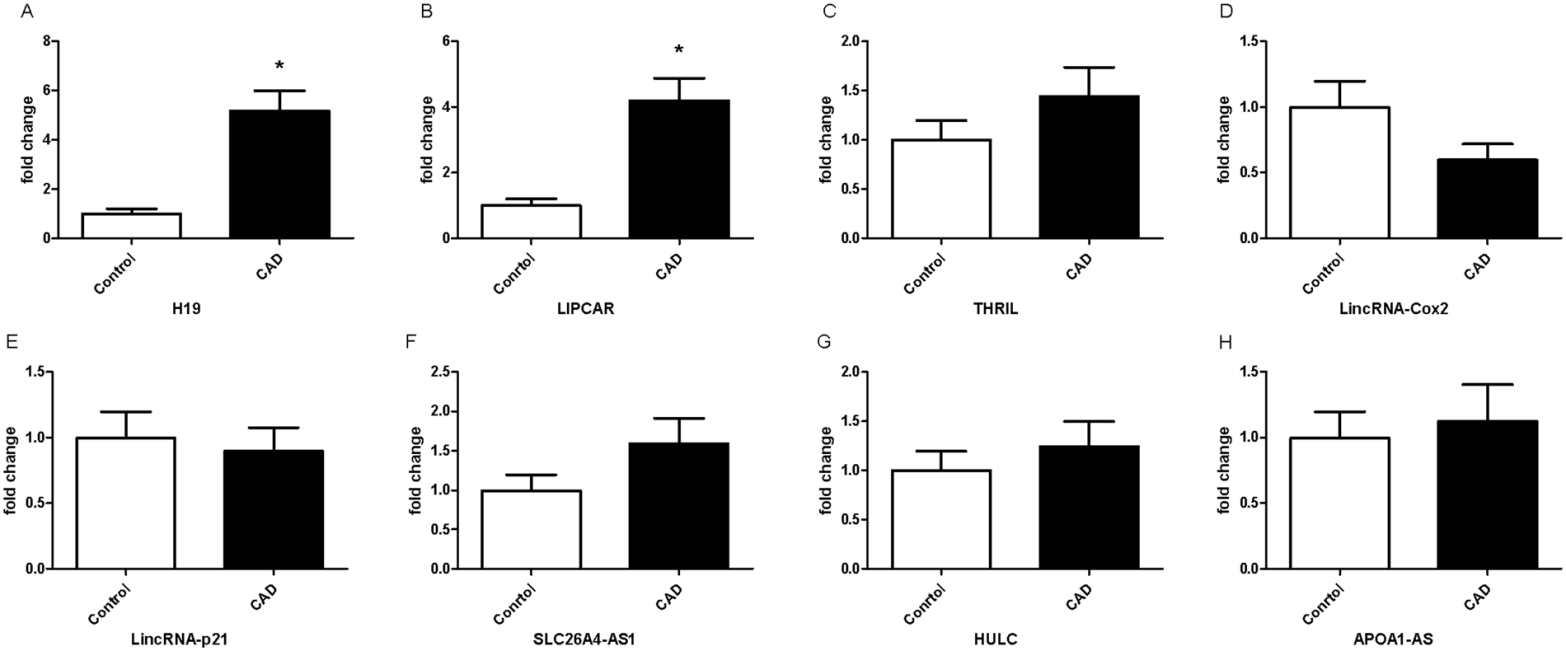
Plasma levels of lncRNAs in patients with CAD and controls. Plasma levels of H19 (**A**) and LIPCAR (**B**) are increased in patients with CAD compared with controls. No significant difference was observed in the plasma levels of THRIL (**C**), LincRNA-Cox2 (**D**), LincRNA-p21 (**E**), SLC26A4-AS1 (F), HULC (**G**), and APOA1-AS (**H**) between patients with CAD and controls. CAD, coronary artery disease; *P < 0.05.

**Figure 2:**
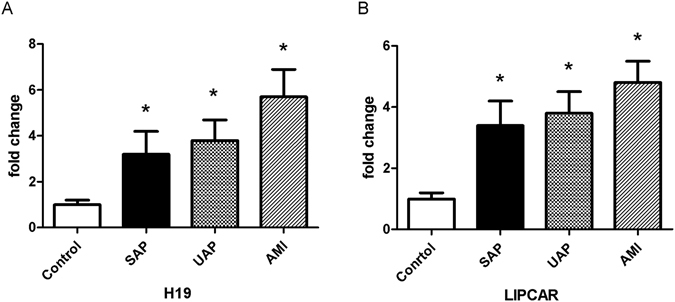
Plasma levels of H19 and LIPCAR in patients with SAP, UAP and AMI. Plasma levels of H19 (**A**) and LIPCAR (**B**) are increased in patients with SAP, UAP and AMI when compared with controls. However, no significant difference was observed among three subgroups. SAP, stable angina pectoris; UAP, unstable angina pectoris; AMI, acute myocardial infarction; *P < 0.05.

Compared with the control group, plasma levels of H19 and LIPCAR were higher in CAD patients (*P*  < 0.001, Figure 1A and B). However, no significant difference was observed in the plasma levels of THRIL, LincRNA-Cox2, LincRNA-p21, HULC, SLC26A4-AS1 and APOA1-AS between patients with CAD and controls (Fig. 1C–H). We further divided CAD patients into three subgroups, including stable angina pectoris (SAP), unstable angina pectoris (UAP), and acute myocardial infarction (AMI). As shown in Fig. 2, patients with AMI appeared to have the highest circulating levels of H19 and LIPCAR, although the difference did not reach statistical significance.

### Correlation between plasma H19 and LIPCAR with clinical characteristics

We further analyzed the correlations of plasma levels of H19 and LIPCAR with clinical characteristics in patients with CAD. As shown in Table 2, plasma levels of H19 was positively associated with BMI (*R* = 0.121, *P* = 0.022), LDL-C (*R* = 0.134, *P* = 0.012), and Gensini score (*R* = 0.161, *P* = 0.003), indicating that increased H19 level may correlated with the severity of CAD. In addition, we also found that the plasma level of LIPCAR was positively associated with age (*R* = 0.201, *P*  < 0.001), but negatively associated with HDL-C (*R* = −0.203, *P* < 0.001).

**Table 2:** Correlations between plasma H19 and LIPCAR with clinical characteristics.

Variables	H19		LIPCAR	
	*R*	*P* value	*R*	*P* value
Sex	−0.058	0.275	0.025	0.616
Smoking	−0.083	0.117	−0.085	0.162
Age	0.047	0.376	0.201	<0.001
BMI	0.121	0.022	0.055	0.363
FBG	−0.086	0.108	−0.06	0.336
TC	−0.093	0.084	−0.066	0.292
LDL-C	0.134	0.012	−0.083	0.182
HDL-C	−0.012	0.824	−0.203	<0.001
TG	−0.084	0.119	0.041	0.513
Creatinine	−0.007	0.889	0.012	0.849
Gensini score	0.161	0.003	−0.081	0.133

BMI, body mass index; FBG, fasting blood glucose; TC, total cholesterol; LDL-C, low-density lipoprotein cholesterol; HDL-C, high-density lipoprotein cholesterol; TG, triglyceride.

### Plasma levels of H19 and LIPCAR are independent risk factor for CAD

Univariate and multivariate logistic regression analysis revealed that plasma levels of H19 and LIPCAR were significantly associated with the presence of CAD, even after adjustment for age, gender, BMI, smoking, hypertension, diabetes, and blood lipid profiles, univariate and multivariate logistic regression analysis revealed that plasma levels of H19 and LIPCAR were significantly associated with the presence of CAD (Table 3).

**Table 3:** Univariate analysis and multiple logistic regression analysis for the risk of CAD.

Models	OR	95% CI	*P* value
H19			
Univariate analysis	1.089	1.024–1.159	0.007
Multiple logistic regression model^1^	1.089	1.014–1.168	0.018
Multiple logistic regression model^2^	1.107	1.027–1.194	0.008
Multiple logistic regression model^3^	1.095	1.018–1.177	0.015
LIPCAR			
Univariate analysis	1.255	1.143~1.378	0.004
Multiple logistic regression model^1^	1.244	1.128~1.222	0.012
Multiple logistic regression model^2^	1.233	1.108~1.373	0.011
Multiple logistic regression model^3^	1.201	1.065~1.345	0.005

The model^1^ included age, gender, BMI, and smoking.

The model^2^ included age, gender, BMI, smoking, hypertension, and diabetes.

The model^3^ included age, gender, BMI, smoking, hypertension, diabetes, TC, TG, LDL-C, and HDL-C.

OR, odds ratio; CI, confidence interval.

### Stratification analyses of the plasma levels of H19 and LIPCAR with the risk of CAD

Stratified analyses were conducted according to gender, age, diabetes and smoking status. As shown in Table 4, we found that the predictive effect of plasma H19 on the risk of CAD was more prominent in females (Adjusted OR = 1.126, 95% CI = 1.021–1.241, *P* = 0.017), elderly (Adjusted OR = 1.115, 95% CI = 1.006–1.236, *P* = 0.039) and non-diabetic subjects (Adjusted OR = 1.092, 95%CI = 1.013–1.177, *P* = 0.021). While for LIPCAR, as shown in Table 5, the predictive effect on the risk of CAD was more prominent in younger subjects (Adjusted OR = 1.306, 95% CI = 1.061–1.607, *P* = 0.012), non-diabetic subjects (Adjusted OR = 1.227, 95%CI = 1.090–1.382, *P* = 0.001), and non-smoking subjects (Adjusted OR = 1.682, 95% CI = 1.198–2.361, *P* = 0.003).

**Table 4:** Stratification analyses of plasma levels of H19 with the risk of CAD.

Variables	Univariate analysis		Multiple logistic regression model^1^		Multiple logistic regression model^2^		Multiple logistic regression model^3^	
	OR (95%CI)	*P* value	Adjusted OR (95%CI)	*P* value	Adjusted OR (95%CI)	*P* value	Adjusted OR (95%CI)	*P* value
Sex								
Females	1.092 (1.014–1.175)	0.020	1.109 (1.016–1.210)	0.021	1.120 (1.024–1.226)	0.013	1.126 (1.021–1.241)	0.017
Males	1.087 (0.958–1.233)	0.195	1.008 (0.884–1.149)	0.910	1.048 (0.905–1.213)	0.531	1.034 (0.902–1.186)	0.630
Age								
<60 years	1.095 (0.977–1.226)	0.118	1.095 (0.977–1.227)	0.118	1.098 (0.980–1.230)	0.106	1.111 (0.988–1.250)	0.079
≥60 years	1.084 (1.002–1.171)	0.043	1.084 (0.994–1.183)	0.068	1.085 (0.995–1.182)	0.067	1.115 (1.006–1.236)	0.039
Diabetes								
No	1.097 (1.023–1.178)	0.010	1.081 (1.008–1.159)	0.029	1.098 (1.020–1.182)	0.013	1.092 (1.013–1.177)	0.021
Yes	2.061 (0.365–11.642)	0.413	2.206 (0.382–12.743)	0.377	1.886 (0.453–7.851)	0.384	1.896 (0.163–22.088)	0.610
Smoking								
No	1.116 (1.000–1.246)	0.050	1.105 (0.991–1.231)	0.073	1.106 (0.988–1.237)	0.079	1.110 (0.985–1.250)	0.087
Yes	1.066 (0.991–1.147)	0.084	1.075 (0.983–1.177)	0.114	1.103 (0.999–1.218)	1.103	1.101 (0.994–1.219)	0.066

The model^1^ included age, gender, BMI, and smoking.

The model^2^ included age, gender, BMI, smoking, hypertension, and diabetes.

The model^3^ included age, gender, BMI, smoking, hypertension, diabetes, TC, TG, LDL-C, and HDL-C.

OR, odds ratio; CI, confidence interval.

**Table 5:** Stratification analyses of plasma levels of LIPCAR with the risk of CAD.

Variables	Univariate analysis		Multiple logistic regression model^1^		Multiple logistic regression model^2^		Multiple logistic regression model^3^	
	OR (95%CI)	*P* value	Adjusted OR (95%CI)	*P* value	Adjusted OR (95%CI)	*P* value	Adjusted OR (95%CI)	*P* value
Sex								
Females	1.239 (1.110–1.383)	0.015	1.241 (1.112–1.384)	0.001	1.229 (1.094–1.381)	0.012	1.208 (1.050–1.389)	0.008
Males	1.282 (1.079–1.523)	0.005	1.305 (1.094–1.555)	0.003	1.285 (1.027–1.606)	0.028	1.347 (1.042–1.740)	0.023
Age								
<60 years	1.291 (1.113–1.497)	0.001	1.282 (1.104–1.488)	0.001	1.277 (1.090–1.497)	0.003	1.306 (1.061–1.607)	0.012
≥60 years	1.201 (1.066–1.353)	0.003	1.222 (1.083–1.379)	0.001	1.210 (1.042–1.406)	0.013	1.181 (1.010–1.380)	0.037
Diabetes								
No	1.248 (1.136–1.372)	0.002	1.241 (1.125–1.370)	0.129	1.228 (1.103–1.367)	0.001	1.227 (1.090–1.382)	0.001
Yes	1.350 (0.835–2.183)	0.220	1.314 (0.793–2.178)	0.289	1.302 (0.772–2.163)	0.245	1.341 (0.776–2.172)	0.256
Smoking								
No	1.388 (1.134–1.699)	0.001	1.439 (1.156–1.791)	0.001	1.463 (1.151–1.860)	0.002	1.682 (1.198–2.361)	0.003
Yes	1.208 (1.081–1.350)	0.014	1.162 (1.035–1.304)	0.011	1.160 (1.021–1.317)	0.022	1.120 (0.983–1.275)	0.089

The model^1^ included age, gender, BMI, and smoking.

The model^2^ included age, gender, BMI, smoking, hypertension, and diabetes.

The model^3^ included age, gender, BMI, smoking, hypertension, diabetes, TC, TG, LDL-C, and HDL-C.

OR, odds ratio; CI, confidence interval.

### Evaluation of plasma H19 and LIPCAR as novel biomarkers for CAD

**Figure 3:**
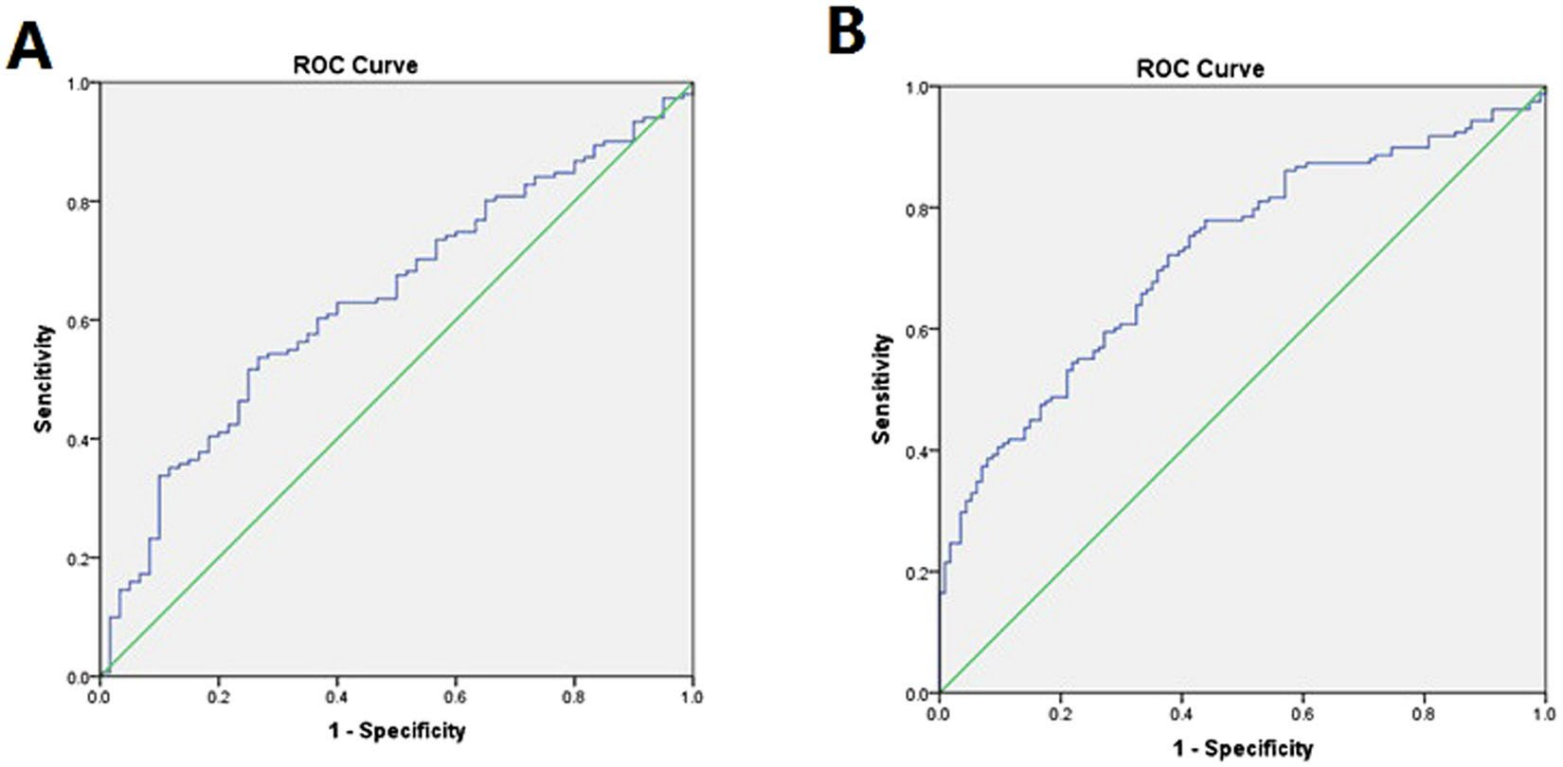
The receiver operating characteristic (ROC) curve analyses for the plasma H19 and LIPCAR as diagnostic biomarkers of CAD. The area under ROC curve (AUC) was 0.631 (95% CI = 0.551–0.788) for H19 (**A**) and 0.722 (95% CI = 0.669–0.782) for LIPCAR (**B**). The sensitivity and specificity at the optimal cut-off were 53.6% and 73.0% for H19 (cut-off value: 0.269), and 72.2% and 62.3% for LIPCAR (cut-off value: 0.344), respectively.

Having established that plasma H19 and LIPCAR are independent risk factors for CAD, we sought to determine the potential utility of plasma H19 and LIPCAR as diagnostic biomarkers of CAD. To this end, ROC analysis was performed to evaluate the predictive power of plasma H19 and LIPCAR for HF. Our results showed that the area under ROC curve (AUC) was 0.631 (95% CI = 0.551–0.788) for H19 (Fig. 3A) and 0.722 (95% CI = 0.669–0.782) for LIPCAR (Fig. 3B). The sensitivity and specificity at the optimal cut-off were 53.6% and 73.0% for H19, and 72.2% and 62.3% for LIPCAR, respectively.

### Plasma levels of H19 and LIPCAR are increased in CAD patients with chronic heart failure (CHF)

**Figure 4:**
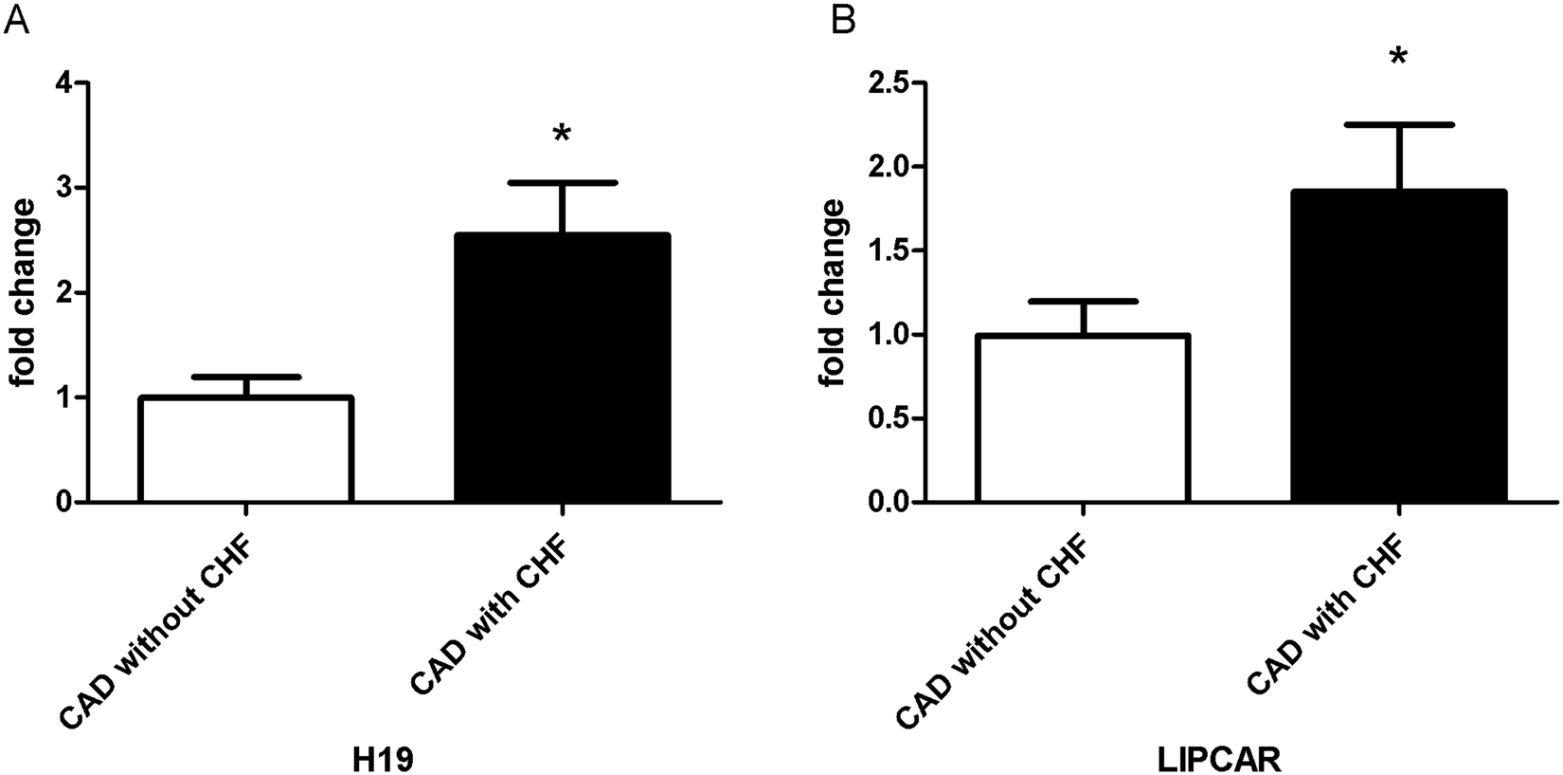
Plasma levels of H19 and LIPCAR in CAD patients with and without CHF. Plasma levels of H19 (**A**) and LIPCAR (**B**) are increased in CAD patients with CHF when compared to those without CHF. CAD, coronary artery disease; CHF, chronic heart failure; *P < 0.05.

Since both H19 and LIPCAR have been demonstrated to be involved in the pathological process of heart failure^19, 26^, we further investigated the differences of these two lncRNAs between CAD patienrs with and without CHF. As shown in Fig. 4, plasma levels of H19 and LIPCAR were both higher in patients with CHF (*P* = 0.014 for H19; *P* = 0.038 for LIPCAR, respectively).

## Discussion

In the present study, we investigated the plasma levels of a selected set of cardiac-related or atherosclerosis-related lncRNAs for their potential as novel biomarkers of coronary artery disease (CAD). We here showed that the plasma levels of two lncRNAs, H19 and LIPCAR were significantly increased in patients with CAD. Multivariate logistic regression analysis revealed that plasma levels of H19 and LIPCAR were independently associated with the risk of CAD, even after adjustment for traditional cardiovascular risk factors. Our results indicated that plasma H19 and LIPCAR might be served as promising candidate biomarkers for CAD.

The lncRNA H19 is a well-known imprinted gene which is abundantly expressed from the early stage of embryogenesis throughout fetal life, but is downregulated postnatally^27^. Accumulating data indicate that re-expression of H19 may play important roles in cardiovascular diseases^28, 29^. The expression levels of H19 were increased in human VSMCs treated with homocysteine and aortae of mice with hyperhomocysteinemia, which is a well-known independent risk factor for CAD^30, 31^. A recent study showed that over-expression of H19 could promote atherosclerosis by activating MAPK and NF-κB signaling pathway^32^. Our previous study also demonstrated that the common polymorphisms of H19 were associated with the risk and severity of CAD^33^. We here found that increased plasma level of H19 was independently correlated with the risk of CAD and positively associated with the severity of CAD evaluated by the Gensini score. Our stratified analysis revealed that the increased risk of CAD associated with the plasma levels of H19 was more prominent in subgroups of females, elderly, non-diabetic subjects. The most possible explanation for this sex and age difference is that as an imprinted gene, paternal allele of H19 is imprinted, while only the maternal allele is expressed^34^. Moreover, H19 is located in close proximity to the insulin-like growth factor 2 (IGF2) gene on human chromosome 11p15.5 and can downregulate the expression of IGF2 in *cis* and *trans* ^35^. IGF2 is one the key regulators in insulin signaling and is involved in the development of diabetes^36^. Thus, we speculated that abnormal insulin regulation in diabetes may also affect the predictive role of plasma H19 for CAD in our study. However, further studies are needed to confirm this hypothesis.

Another interesting finding in our study is that plasma H19 is increased in CAD patients with chronic heart failure (CHF). Our results are consistent with a recent study which showed that cardiac H19 level was increased in ischemic end-stage failing hearts^37^. Liu *et al*. showed that H19 could regulated the development of cardiac hypertrophy through miR-675/CaMKIIδ pathway^26^, indicating that dysregulation of H19 may also contribute to the pathogenesis of ischemic heart failure.

LIPCAR is considered as a mitochondria-derived lncRNA which can be readily detected in circulating^19^. Circulating level of LIPCAR is upregulated in patients with CHF independently of the pathogenesis, and that higher LIPCAR levels were associated with a higher risk of cardiovascular death^19^. We here also found that increased plasma LIPCAR level was independently correlated with increased risk of CAD. Further analyses showed that plasma LIPCAR levels were higher in CAD patients with CHF when compared to those without CHF. Our results are consistent with another study which demonstrated that circulating LIPCAR levels inversely correlated with echocardiographic E/A ratio, which is marker of LV dysfunction^38^. Our stratified analysis revealed that the increased risk of CAD associated with the plasma levels of LIPCAR was more prominent in subgroups of younger, non-diabetic, and non-smoking subjects. De Gonzalo-Calvo, D. *et al*. also showed that circulating LIPCAR is associated with left ventricular diastolic function and remodeling in patients with well-controlled type 2 diabetes, even after adjustment for possible confounding factors^38^. The mechanism underlying the correlation of LIPCAR with CAD remains unclear. It has been found that LIPCAR is strongly correlated with waist circumference, plasma fasting insulin, subcutaneous fat volume and HDL-C^19^. Our data also demonstrated that plasma level of LIPCAR was negatively associated with HDL-C. Thus, increased LIPCAR may induce metabolic dyshomeostasis, which in turn promotes atherosclerosis. Moreover, mitochondrial dysfunction is implicated in the etiology of cardiovascular diseases, especially CAD^38^. Thus, as a mitochondria-derived lncRNA, a potential function of LIPCAR in regulating mitochondrial pathways, such as oxidative phosphorylation and inflammasome activation, needs to be explored in future studies transcriptional.

Other limitations may also be addressed. Firstly, selection bias in the present study might have affected our results. Large-scale, multicenter studies are required to further elucidate the role of H19 and LIPCAR as a potential biomarker for the risk of CAD. Secondly, the underlining mechanisms of the association between up-regulated H19 and the severity of CAD need to be further studied. Thirdly, further experimental studies using animal or cellular model are needed to explore the mechanisms by which H19 and LIPCAR participate in the development of atherosclerosis.

## Conclusion

In conclusion, our study showed that plasma levels of H19 and LIPCAR are associated with increased risk of CAD and may be considered as novel biomarkers for CAD.

## Electronic supplementary material

Supplementary Information
