# Supplementary material for: Increased plasma levels of lncRNA H19 and LIPCAR are associated with increased risk of coronary artery disease in a Chinese population
Source: Sci Rep. 2017 Aug 8;7:7491. doi: 10.1038/s41598-017-07611-z (PMC5548926; doi:10.1038/s41598-017-07611-z)
Supplement: Supplementary file 1 — Supplementary Information [file 41598_2017_7611_MOESM1_ESM.docx]

**Increased plasma levels of lncRNA H19 and LIPCAR are associated with increased risk of coronary artery disease in a Chinese population**

Zhen Zhang ^a^**^,†^**, Wei Gao ^b^**^,†^**, Jian Zhang ^a,c,^**^†^**, Ya-Fei Li ^a^, Qing-Qing Long ^a^, Dong-Chen liu ^a^, Jian-Jun Yan ^a^, Zhi-Jian Yang ^a,^*, Lian-Sheng Wang ^a,^*

^a^ Department of Cardiology, the First Affiliated Hospital of Nanjing Medical University, Nanjing, 210029, China.

^b^ Department of Geriatrics, Sir Run Run Hospital, Nanjing Medical University, Nanjing, 211166, China.

^c^ Department of Cardiology, Sir Run Run Hospital, Nanjing Medical University, Nanjing, 211166, China.

^†^ There authors contributed equally to this work.

*Corresponding authors: Lian-Sheng Wang, MD, PhD, Department of Cardiology, the First Affiliated Hospital of Nanjing Medical University, 300 Guangzhou Road, Nanjing, 210029, Jiangsu Province, China. E-mail: drlswang@njmu.edu.cn, Tel./Fax: 0086-25-83724440; Zhi-Jian Yang, MD, PhD, Department of Cardiology, the First Affiliated Hospital of Nanjing Medical University, 300 Guangzhou Road, Nanjing, 210029, Jiangsu Province, China. E-mail address: [zhijianyangnj@yahoo.com.cn](mailto:zhijianyangnj@yahoo.com.cn), Tel./Fax: 0086-25-83724440.

**Supplemental Table 1. Primers for the qRT-PCR**

| Gene | Forward primer | Reverse primer |
| --- | --- | --- |
| H19 | AGAAGGCTGGGGCTCATTTG | GCAGGAGGCATTGCTGATGAT |
| LIPCAR | TAAAGGATGCGTAGGGATGG | TTCATGATCACGCCCTCATA |
| APOA1-AS | GCAAACCTTCTTCATCCACCAG | GGCACGCTAGTCTGTTGAGTA |
| THRIL | TTGGTTGTAGCAGGTCTGGC | GGCAACAGAGCAAGACTTCATC |
| LincRNA-Cox2 | TGGAGGAGTCAGGAGGAATAGG | GCTGGCATGGACAAGTTGAAG |
| LincRNA-p21 | GGGTGGCTCACTCTTCTGGC | TGGCCTTGCCCGGGCTTGTC |
| SLC26A4-AS1 | TGAAAGGCAGAAGGAAGGGTTT | AAGGCAGGTGGATTACGGAAG |
| HULC | CAACCTCCAGAACTGTGATCCA | CTTGCTTGATGCTTTGGTCTGT |
| cel-miR-39 | Catalog # 4427975(Invitrogen) | |
